# Miscellaneous

**Published:** 1874-02

**Authors:** 


### MISCELLANEOUS.

ABOUT BEES.—The king is stingless, "armed only with his majestic port." Modern science regards this so-called king as mother and monarch of the hive. It is found that she lives four years, and is hatched from the egg in fourteen days, while the workers require twenty-one days, and the drones twenty-four. These strange figures are part of the mystery attaching to bees; but a still more curious fact connected with this point is that the bees have the power at will of developing common eggs into queen bees. This is done by removing an egg into a royal cell, and feeding the little grub with a substance of a milky, gelatinous appearance known as "royal jelly." These facts have been ascertained without doubt by Mr. Pittigrew, one of the most successful bee keepers of the day; though what the exact analysis of this "royal jelly" may be is utterly unknown. The chief function of the queen in the hive is to lay eggs, from which the future population will spring. A healthy queen during her life is estimated to lay the enormous number of 800,000. Often in the heat of summer, for two months together, she will lay 2,000 a day. Whether these eggs are all alike, or whether some are distinctly worker eggs, is one of the numerous questions on which all bee keepers are at issue. The working bees form the life and prosperity of the hive. To them belong industry, labor, patience, ingenuity—in short, all the virtues of the race; and while each knows his own duty, and does it, the efforts of all are directed toward the weal of the community. The working bee never lives longer than nine months; they labor so incessantly that it is supposed they never sleep. The daily consumption and waste of a large hive of bees in summer may be taken at two pounds of honey; it will show the industry of the working bees to bear in mind that beyond this, such a hive in favorable weather will often accumulate honey to the amount of four and six pounds daily. Indeed, it is upon record that a hive once gained twenty pounds weight of it in two days. It is curious that even a wild hive of bees can soon be taught to recognize and refrain from attacking people who approach them. No wonder that the ancients esteemed them divine; that their poet laureate, according to the Platonic philosophy, assigns them "a participation in the supreme mind and the heavenly influences," and that another speaks of their powers of presaging wind and fine weather.

BORAX FOR COLDS.—A writer in *The Medical Record* cites a number of cases in which borax has proved a most effective remedy in certain forms of colds. He states that in sudden hoarseness or loss of voice in public speakers or singers, from colds, relief for an hour or so, as by magic, may be often obtained by slowly dissolving, and partially swallowing a lump of borax the size of a garden pea, or about three or four grains, held in the mouth for ten minutes before speaking or singing. This produces a profuse secretion of saliva, or "watering" of the mouth and throat—probably restoring the voice or tone to the dried vocal cords, just as "wetting" brings back the missing notes to a flute, when it is too dry.

NUTRITIVE VALUE OF WHEAT MEAL.—*The London Dietetic Reformer* shows, by scientific data, that wheat meal, which is cheaper than bolted meal or fine flour, contains one third more nutriment than flour does, from which the bran has been sifted. Fine flour, according to this journal, is not food at all, in the proper sense of the term—that is, the elements of the grain which are separated in the process of bolting, being essential to perfect nutrition, those who use fine flour are obliged to subsist mainly on other things, or lose their health; that no one, therefore, who makes baker's bread a principal article of diet can long maintain health, while those who use wheat meal bread, unfermented and unadulterated, can maintain their health with a very small addition of other foods.

Another Theory Concerning Dreams.—A novel and interesting theory as to the nature of dreams was lately propounded before the Royal Medical Institution, by Prof. Humphrey, of Cambridge, England, defining dreams as not a normal accompaniment of sleep, but rather a result of the abnormal or imperfect condition of the organ of mental action. In the natural state, he says, we should pass from wakefulness to complete unconsciousness, and *vice versa*, almost instantaneously, and this is the case with many persons; more frequently, however, the transition is protracted. and stages are observed in which the sleep is but partial, In this case, according to Prof. Humphrey, the cerebral organ being in an imperfect state, its action is imperfect, and the first effect of the lessening of its vital vigor is a loss of the highest form of mental power—the control over the mental operations; and, in this condition, the thoughts ramble unchecked, chase one another confusedly over the mental field, and give rise to all sorts of incongruities of the imagination.

Sea Sickness.—The opinion so commonly held in regard to sea sickness, namely, that it is due either to a congestion of the brain or to a commotion in the abdominal viscera, caused by the motion of the vessel, are very plausibly combatted by M. Pellerin, who, in a paper read before the French Academy, attributes the malady to a deranged circulation of the blood, produced by the alternate rolling and heaving of the vessel. The result of this, he says, is not a congestion of the brain, which is, on the contrary, deprived of some of the blood required to keep up a stimulus of that nervous centre—that sensation which is felt in sea sickness resembling peculiarly what is felt immediately after a letting of the blood when the patient sits or stands, namely, a disposition to vomit, or actual vomiting. In support of this opinion, mention is made of the fact that persons who are liable to sea sickness experience its effects in a much slighter degree when they are in a horizontal position—the relief thus afforded being like that which is produced in the same position when a person is in a state of syncope.

THE KITCHEN AS THE ARBITER OF HUMAN DESTINIES.—Dr. Edward Jarvis in his paper before the American Public Health Association, on "The Housekeeper's Responsibility," said:

"It depends upon the kitchen whether the family shall be robust, bright and energetic, or dull, stupid and slow. The housekeeper measures out manhood and womanhood to the family, and her position is then a highly responsible one. There is here field for the display of talent and discretion. The employments of men may offer less scope for the adaptation of great ideas to great ends than the housekeeper's. Woman is not a housekeeper by instinct or cook by nature any more than men are natural shoemakers, but she has the capacity for the former work which he may have for the latter. They cannot be perfect in the duties of a housekeeper without suitable preparation any more than they can be milliners. Boys are trained to their professions. No such training is given the daughters for that station which is admitted to be their high aim in life—the superintendence of the household. The result of carelessness in the kitchen is styled 'ill luck.' It is 'unlucky' that the bread is heavy. A carpenter might as well say it is unlucky that his window beams are too short. Nowhere except in the kitchen, where so much depends upon care in selection and preparation, is the use of the measure done away with: nowhere is so little care given to the selection of the articles of purchase. Ribbons are turned over and over to find a certain color, if some trimming for a dress is required, but the servant is sent to market to get a few pounds of any kind of beef for the table."

THE SLEEP OF PLANTS.—The phenomenon called the sleep of plants was first observed by the illustrious Swedish botanist, Linnæus. He remarked in the bird's foot trefoil the difference between the attitude of the leaves during the day and night. He almost immediately concluded that this would prove to be a general phenomenon in vegetable life. Continuing his observations, Linnæus soon satisfied himself that this change in the position of leaves during the night is observable in many vegetables. He regarded the absence of light, and not the nocturnal cold, as the principal cause of the phenomenon; for plants in hot houses close themselves during the night like those in the open air. He found the difference between waking and sleeping to be much less apparent in young plants than in more matured ones—being most clearly indicated among the compound leaves.

